# The safety of selective COX-2 inhibitors in tonsillectomy patients: a systematic review

**DOI:** 10.1017/S0022215126105039

**Published:** 2026-08

**Authors:** Sophie Dunmall, Lucylynn Lizarondo, Romy Jia, Eng Hooi Ooi

**Affiliations:** 1 Flinders Medical Centre, Australia; 2 JBI Adelaide, Australia; 3 Otolaryngology Head and Neck Surgery, Flinders Medical Centre, Australia

**Keywords:** analgesia, Tonsillectomy, hemorrhage, cyclooxygenase 2 inhibitors, hemorrhage, non-steroidal anti-inflammatory agents, analgesiatonsillectomy

## Abstract

**Objectives:**

This study aimed to determine the safety of selective cyclooxygenase 2 inhibitors in tonsillectomy patients.

**Methods:**

A systematic review of the literature was undertaken in August 2024 (updated March 2025) using Joanna Briggs Institute systematic review and critical appraisal methodology. Study outcomes were bleeding incidence, analgesic efficacy and adverse effects. Eligible comparators were non–cyclooxygenase-2-selective non-steroidal anti-inflammatory drugs or placebo.

**Results:**

Fifteen studies were included for meta-analysis, 14 of which were randomised control trials. The studies were overall of good quality. There was no increased risk of bleeding with selective cyclooxygenase 2 inhibitors (odds ratio = 1.27; 95 per cent confidence interval: 0.51 to 3.16; *p* = 0.61). They were more effective than placebo in providing analgesia (odds ratio = -1.22; 95 per cent confidence interval, -2.12 to -0.33; Z = 2.67; *p* = 0.007; I^2^ = 84 per cent).

**Conclusion:**

Selective cyclooxygenase 2 inhibitors do not cause an increase in bleeding risk for tonsillectomy patients. There is insufficient evidence to compare their safety and efficacy to non-selective non-steroidal anti-inflammatory drugs such as ibuprofen.

## Introduction

Tonsillectomy is performed in both adults and children to treat recurrent infections and sleep disordered breathing symptoms due to tonsillar hypertrophy.^1^ Two of the most significant complications of tonsillectomy are pain and bleeding.^2^ Pain can last for up to 12 days post-operatively,^3^ and effective analgesia is required as it helps patients maintain adequate oral intake during the recovery period.^4^^,^^5^ Bleeding can be a serious complication necessitating blood transfusion and return to the operating theatre to arrest severe haemorrhage,^6^ so it is prudent for surgeons to carefully manage risk factors for post-tonsillectomy bleeding.^1^

Minimising opioid use for analgesia after tonsillectomy has the benefits of avoiding undesirable side effects such as sedation, respiratory depression, constipation, and potential for misuse.^7^ Paracetamol offers analgesia with minimal risk; however, it is rarely sufficient when prescribed alone to treat post-tonsillectomy pain.^8^ NSAIDs (non-steroidal anti-inflammatory drugs) can be a useful adjunct in multi-modal analgesia to help limit the use of opiates; however, their use post-tonsillectomy has been historically controversial. Some NSAIDS such as ketorolac have been previously associated with an increased risk of peri-operative bleeding in tonsillectomy.^9^^,^^10^ Given these considerations, understanding the efficacy of pain control and bleeding risk is essential in evaluating peri-operative analgesic strategies for tonsillectomy.

The anti-inflammatory mechanism of NSAIDs is through cyclooxygenase (COX) enzyme inhibition, which in turn limits the production of pro-inflammatory prostaglandins. There are two COX isoforms, COX-1 and COX-2. COX-1 is thought to be expressed throughout the body with gastrointestinal, renal and platelet effects as part of normal physiologic function. By contrast, COX-2 expression is induced in the presence of growth factors and cytokines released during tissue injury and infection,^11^ which makes it a desirable target when treating pain and inflammation.

The irreversible anti-platelet effect of potent COX-1 inhibitors such as aspirin is associated with increased risk of major haemorrhage.^12^ ENT surgeons have been concerned that a similar COX-1 inhibition mechanism in NSAIDS (though reversible) can lead to increased rates of bleeding post-tonsillectomy.^13^ All NSAIDs act on both COX isoforms to some extent; however, the degree to which they select an isoform is expressed as COX selectivity.^14^

Common and older NSAIDs such as ibuprofen, aspirin and indomethacin are non-selective, that is, they acted effectively on both COX-1 and COX-2. More recently, selective COX-2 inhibitors have been developed with the aim of improving analgesic efficacy and decreasing unwanted side effects such as gastric ulcers and bleeding. The degree of COX-2 selectivity of various NSAIDs can be quantified (Table 1).

**Table 1.** Summary of included study characteristics Table 1 long description.

Author, date	Country	Drug (IC80)	Comparator (IC80)	Participants	Age	Study design
Ng, 2017	Australia	Celecoxib	Placebo	80	Adult	RCT
Xie, 2012	China	Parecoxib	Placebo	80	Adult	RCT
Joshi, 2003	USA	Rofecoxib	Placebo	66	Children	RCT
Van Daele, 2016	USA	Celecoxib	Placebo	15	Adult	RCT
Murto, 2015	Canada	Celecoxib	Placebo	195	Children	RCT
So, 2024	Canada	Celecoxib	No celecoxib	4996	Children	Observational cohort study
Giordano, 2023	USA	Celecoxib	Placebo	130	Children	RCT
Nikanne, 2005	Finland	Celecoxib	Ketoprofen/placebo	115	Adult	RCT
Sheeran, 2016	USA	Rofecoxib	Placebo	45	Children	RCT
Louizos, 2006	Greece	Rofecoxib	Ketoprofen	77	Adult	RCT
Aho, 2003	Finland	Nimesulide	Ibuprofen	78	Adult	RCT
Naesh, 2005	New Zealand	Rofecoxib	Placebo	40	Adult	RCT
Li, 2016	China	Parecoxib	Placebo	60	Children	RCT
Lee, 2004	South Korea	Rofecoxib	Ibuprofen	38	Adult	RCT
Lestiansky, 2011	Czech Republic	Etoricoxib	Placebo	75	Adult	RCT

The Clinical Practice Guideline for Tonsillectomy in Children^15^ was published in 2019 by the American Academy of Otolaryngology (AAO-NHS). The guideline strongly recommends ibuprofen, acetaminophen or both for post-operative pain control, based on a high level of confidence in evidence. The guideline cites the systematic review of Riggin *et al*. (2013),^16^ which found no increase in bleeding risk or severity associated with NSAID use in tonsillectomy. However, of the 36 randomised control trials included in the review by Riggin *et al*., only one evaluated a selective COX-2 inhibitor, celecoxib (Nikanne *et al*. in 2005).^17^ Similarly, the guideline references the 2013 Cochrane^18^ review, in which only one of 15 included studies assessed a selective COX-2 inhibitor, rofecoxib (Pickering in 2002).^19^ Although both reviews underpin current recommendations, they provide limited data specific to selective COX-2 inhibitors.

### Systematic reviews on the safety of NSAIDs (regardless of COX isoform selectivity) in tonsillectomy

Earlier systematic reviews examining the broader class of NSAIDs (regardless of COX isoform selectivity) have reported conflicting findings. A 2003 systematic review^20^ of bleeding risk with NSAIDs in tonsillectomy concluded that ketorolac, ibuprofen and ketoprofen increased the risk of post-tonsillectomy bleeding and recommended they not be used for tonsillectomy patients. In contrast, an updated systematic review by Riggin *et al*. 10 years later,^16^ suggested that NSAIDs are safe in paediatric tonsillectomy patients. Similarly, Lewis *et al*.’s Cochrane review^18^ found insufficient evidence to exclude an increased risk of bleeding in paediatric tonsillectomy patients, highlighting persistent uncertainty.

Within the past five years, there have been multiple further systematic reviews on the bleeding risk of ibuprofen specifically with tonsillectomy. Three systematic reviews assessed the bleeding risk of ibuprofen specifically. One review found insufficient quality evidence to support or reject the use of ibuprofen in tonsillectomy patients.^21^ Another found that ibuprofen was safe and effective,^22^ and a third study found there was no dose-dependent bleeding risk with ibuprofen.^23^

### Systematic reviews on the safety of selective COX-2 inhibitors in tonsillectomy

Two systematic reviews on similar topics were identified with a significantly different scope to our review (Stokes *et al*.,^24^ efficacy of selective COX-2 inhibitors in paediatric patients only and Banatwala *et al*.,^25^ efficacy of celecoxib only).

The concern for increased bleeding risk with NSAIDs is a concern in tonsillectomy patients where post-operative bleeding can be a serious and even fatal complication. This systematic review summarises the available evidence for the safety of selective COX-2 inhibitors in tonsillectomy patients and discusses implications for the use of selective COX-2 inhibitors in clinical practice.

## Materials and methods

### Review question

This is the research question this review aimed to investigate: Are selective COX-2 inhibitors safe in tonsillectomy patients?

This question was addressed through a Joanna Briggs Institute (JBI) systematic review of effectiveness and safety, focusing on quantitative evidence. The review was conducted in accordance with the JBI methodology for systematic reviews of effectiveness, incorporating both meta-analysis and narrative synthesis, as appropriate. The systematic review methods were published as a research protocol in the JBI Evidence Synthesis journal in 2025.^26^

### Participants

The review considered studies that included patients of any age undergoing tonsillectomy or adenotonsillectomy. Tonsillectomy performed for any indication and using any surgical technique was eligible for inclusion. Studies were excluded if patients had pre-existing bleeding disorders or were receiving concurrent antiplatelet/anticoagulant medication.

### Intervention

This review considered studies evaluating the safety of selective COX-2 inhibitors. Selective COX-2 inhibitors could be administered intra-operatively or post-operatively, at any dose, via any route and for any duration. Studies in which non-selective NSAIDs were co-administered in the intervention group were excluded.

### Comparator

This review considered studies comparing selective COX-2 inhibitors with either a placebo or conventional (non-selective) NSAIDs, such as ibuprofen. Comparisons with placebo were used to estimate the absolute bleeding risk associated with selective COX-2 inhibitors, while comparisons with conventional NSAIDS allowed assessment of relative safety and efficacy (for example supplemental opioid requirement as a function of analgesic effectiveness). Selective COX-2 inhibitors were defined as agents with an IC80 ratio greater than 5^14^ (where IC80 is the drug concentration at which 80 per cent of a COX enzyme is inhibited).

### Outcomes

The primary outcome of this review was the incidence of post-tonsillectomy bleeding (PTB), expressed as the proportion of patients requiring return to theatre for surgical control of post-operative bleeding.^6^

Secondary outcomes included the number of doses of pro re nata (PRN) or “as needed” opioid analgesia. Additional secondary outcomes, where reported, included readmission for poorly controlled post-operative pain and the incidence of known NSAID-related adverse effects,^27^ such as acute kidney injury, abdominal pain, gastrointestinal bleeding and cardiovascular events.

Studies of any duration of follow-up were eligible for inclusion, provided the follow-up period was sufficient to assess the relevant outcome(s) of interest for which data were included in the meta-analysis.

### Search strategy

The search strategy aimed to locate both published and unpublished studies. A three-step search strategy was utilised in this review. First, an initial limited search of MEDLINE (PubMed) was undertaken to identify articles relevant to the topic. The text words contained in the titles and abstracts of relevant articles, and the index terms used to describe the articles, were used to develop a full search strategy (see Appendix A). The search strategy was developed in consultation with an Adelaide University health sciences librarian. The final search strategy, including all identified keywords and index terms, was adapted for each included database and information source. Finally, the reference lists of all included studies in the systematic review were screened for additional studies (backward citation searching).

Studies published in any language and on any date were eligible for inclusion. Articles published in languages other than English were screened using DeepL translation tool.

The databases searched included CINAHL (EBSCOhost), the Cochrane Library, Embase (Ovid), MEDLINE (Ovid), Scielo and Web of Science. Sources of unpublished studies and grey literature included the World Health Organization’s International Clinical Trials Registry Platform, the Networked Digital Library of Theses and Dissertations, CAPES theses banks and SCOPUS.

### Study selection

Following the search, all identified citations were collated and uploaded into Covidence. A pilot test was conducted by two reviewers on 10 studies prior to the title and abstract screening, and five studies before the full text review. Following this, titles and abstracts were screened by two independent reviewers (S.T. and R.J.) for assessment against the inclusion criteria. Finally, the reviewers (S.T. and R.J.) assessed the full texts of the selected articles against the inclusion criteria. Any disagreements between reviewers at each stage of the selection process were resolved through discussion and/or consultation with the senior reviewer (L.L.).

### Critical appraisal

Eligible studies were critically appraised by two independent reviewers (S.T. and R.J.) using standardised critical appraisal instruments from JBI for experimental and observational studies. All studies, regardless of methodological quality, were included in data extraction and synthesis (where possible).

### Data extraction

Data were extracted from studies included in the review by the reviewers (S.T. and R.J.) using the standardised JBI data extraction tool.

### Data synthesis

Studies were pooled in statistical meta-analysis using Revman^28^ (Review Manager) software. Effect sizes were expressed as either odds ratios (for dichotomous data) and weighted (or standardised) final post-intervention mean differences (for continuous data) and their 95 per cent confidence intervals (CIs) calculated for analysis. The choice of model (random or fixed effects) and method for meta-analysis was based on the guidance by Moola *et al*.^29^ Subgroup analyses of patient age and individual study drug type were conducted where there was sufficient data to investigate. Sensitivity analyses were conducted to test decisions made regarding analysis methods. Heterogeneity was assessed statistically using the standard chi-squared and I-squared tests.

### Establishing certainty in the evidence

The Grading of Recommendations, Assessment, Development and Evaluation (GRADE) approach for grading the certainty of evidence was followed, and a Summary of Findings (SoF) was created using GRADEpro GDT 2013 (McMaster University, ON, Canada).

### Deviation from a priori protocol

The study methods have deviated from the *a priori* protocol in two aspects. First, the classification of COX-2 selectivity was revised. Initially, all drugs more selective for COX-2 than COX-1 (i.e., IC50 ratio > 1) were included. However, this captured multiple commonly used NSAIDs, such as diclofenac, ibuprofen and ketoprofen, which are clinically considered non-selective and were frequently used as comparators to more definitively COX-2 selective NSAIDs. Therefore, Warner’s^14^ stricter definition of COX-2 selectivity (IC80 ratio >5) was used. A list of all relevant NSAIDs identified in the literature search, along with their IC80 values, is provided in Appendix B.

Second, the minimum follow-up period of 2 weeks post-operatively was adjusted. Studies in which a COX-2 inhibitor was administered before or during the operation often followed patients for only 24 hours. Whilst this duration does not capture overall or secondary PTB, the incidence of primary bleeding remains an accurate and clinically relevant measure and was therefore included.

## Results

### Search results

**Figure 1.**
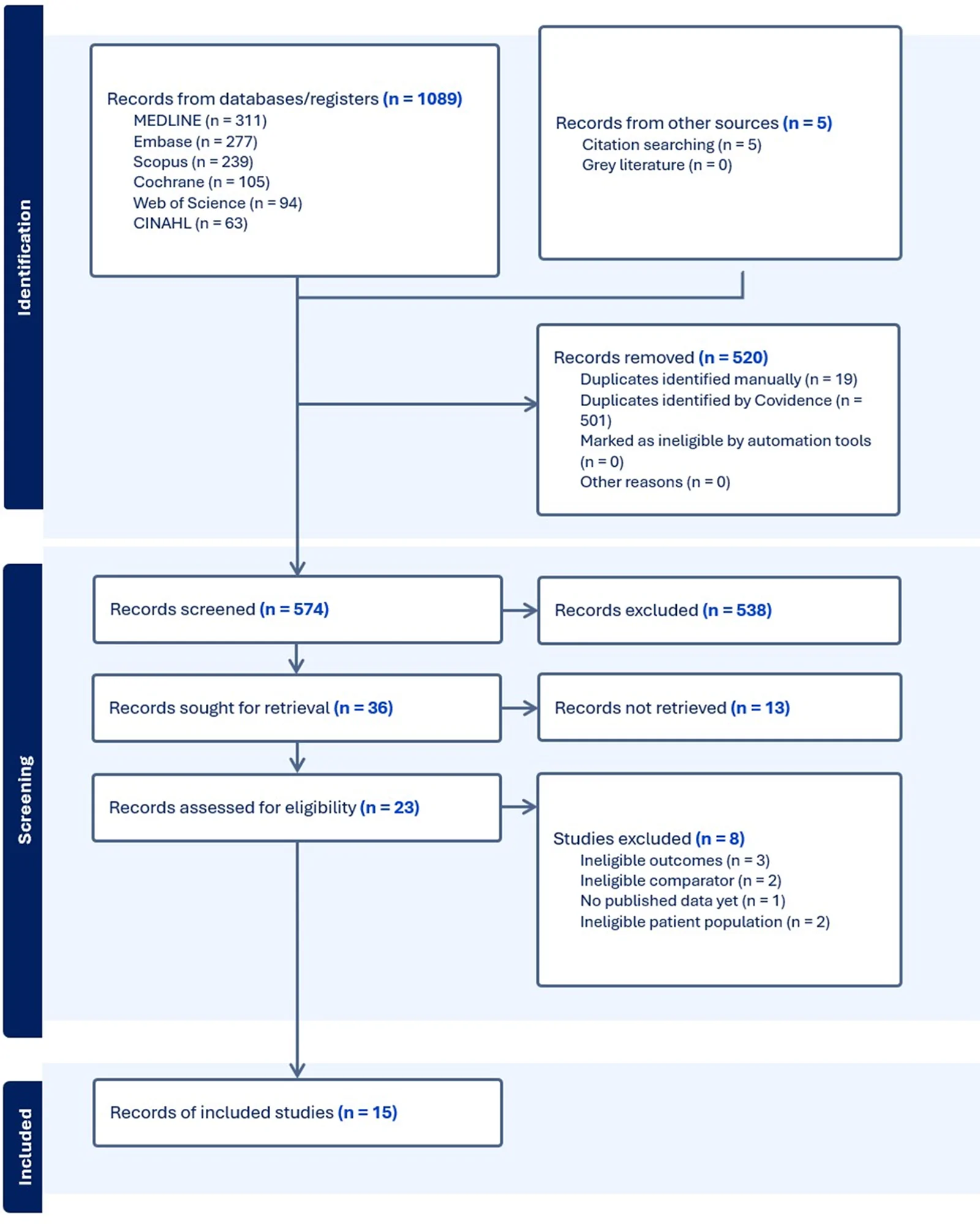
Prisma flow diagram of search results.

The initial database search, conducted on 18 August 2024, identified 1,089 potentially relevant records (Figure 1), with an additional five records identified through citation searching. The search was repeated to capture new studies on 13 March 2025. Seventeen new studies were identified on this additional search; however, none met inclusion criteria for the systematic review.

A total of 15 studies met the inclusion criteria and were included in this review.^17^^,^^30^^–^^43^

### Characteristics of included studies

All included studies except one were randomised control trials (Table 1). The identified selective COX-2 inhibitors (in order of frequency) are celecoxib, rofecoxib, parecoxib, etoricoxib and nimesulide. Placebo was the most common comparator, used in ten studies, while the remaining four prospective studies compared selective COX-2 inhibitors with non-selective NSAIDs such as ibuprofen or ketoprofen. Most studies used an additional analgesic regimen consisting of paracetamol and opiates.

Eight studies^17^^,^^31^^–^^33^^,^^38^^–^^41^ administered the study drug during the patients’ hospital stay for surgery (peri-operative) which offered the opportunity for intravenous or intramuscular medication administration in three of these studies (Table 2). Two studies^35^^,^^43^ administered the study drug both in hospital setting and following discharge, while the remaining five studies^30^^,^^34^^,^^36^^,^^37^^,^^42^ administered the intervention only in the home setting.

**Table 2.** Summary of intervention characteristics and surgical context Table 2 long description.

Author, date	Setting	Indication	Surgeon experience	Surgical method	Drug route	Drug dose/day	Total drug dose
Ng, 2017	Post-discharge	Recurrent infection	Experienced surgeon	Electrocautery	Oral	400mg	2800mg
Xie, 2012	Peri-operative	Recurrent infection	Experienced surgeon	Not stated	Intravenous	80mg	80mg
Joshi, 2003	Peri-operative	Not stated	Not stated	Not stated	Oral	2mg/kg	2mg/kg
Van Daele, 2016	Peri-operative and post discharge	Recurrent infection	Not stated	Electrocautery dissection	Oral	400mg	4600mg
Murto, 2015	Post-discharge	Not stated	Not stated	Not stated	Oral	6mg/kg	21mg/kg
So, 2024	Post-discharge	Obstruction	Experienced surgeon	Monopolar electrocautery	Oral	6mg/kg	51mg/kg
Giordano, 2023	Post-discharge	Not stated	Not stated	Monopolar electrocautery	Oral	12mg/kg	132mg/kg
Nikanne, 2005	Peri-operative	Not stated	Experienced surgeon	Electrocautery dissection	Oral	200mg	200mg
Sheeran, 2016	Peri-operative	Not stated	Not stated	Not stated	Oral	1mg/kg	1mg/kg
Louizos, 2006	Peri-operative	Not stated	Not stated	Not stated	Oral/Intramuscular	50mg	50mg
Aho, 2003	Peri-operative and post discharge	Not stated	Not stated	Electrocautery dissection	Oral	200mg	1600mg + PRN doses
Naesh, 2005	Peri-operative	Not stated	Not stated	Cold steel	Oral	50mg	50mg
Li, 2016	Peri-operative	Recurrent infection and obstruction	Not stated	Cold steel	Intravenous	1mg/kg	1mg/kg
Lee, 2004	Post-discharge	Recurrent infection	Not stated	Electrocautery	Oral	50mg	350mg
Lestiansky, 2011	Peri-operative	Recurrent infection	Experienced surgeon	Cold steel	Oral	120mg	120mg

### Methodological quality of included studies

Overall, the studies were of good methodological quality. Appendix C summarises the critical appraisal outcomes using the JBI critical appraisal tools.^44^^,^^45^ The cohort study critical appraisal tool^44^ was used for So *et al*.,^42^ and the results are described narratively.

True randomisation was mostly well described. Four studies^30^^,^^32^^,^^38^^,^^41^ did not include the specific method of randomisation in their reporting. Blinding was well described in 10 out of the 14 included RCTs. Two studies^30^^,^^39^ did not blind participants or those delivering the treatment. Lee *et al*.^30^ did not describe any blinding method of participants, administrators (i.e., those delivering the treatment) or outcome assessors. Whilst Sheeran *et al*.^38^ described the blinding method for participants, it was unclear if administrators or outcome assessors were adequately blinded. Joshi *et al*.^32^ described blinding for those delivering treatment but not participants. Treatment groups overall were treated identically at baseline.

Bias related to outcome measurement reliability was present in almost half of the included studies. This was due to the nature of attempting to capture analgesic efficacy in patients who had been discharged home in studies with longer follow-up periods. Data collection for these studies relied on self-reported outcomes, either by diary or by recall of events and interview by outcome assessors. It would be impractical for the patient to remain in a hospital setting throughout the entire recovery period, and it would not reflect the typical recovery environment for patients after a tonsillectomy. Seven studies relied on self-reported outcome measures, which carry an associated risk of bias. Follow-up was adequately described and analysed in all but three studies.^30^^,^^32^^,^^41^

Trial design was considered appropriate in most studies. Due to the exposure re-allocation affecting multiple outcomes in Nikanne *et al*.’s^17^ study, the RCT design was considered inappropriate. It was unclear if another four studies^30^^,^^32^^,^^38^^,^^39^ had appropriate trial design, as they inadequately described blinding and follow-up processes.

### Summary of findings across studies

NB asterisk in Forest plot figures denotes studies which have a non-selective NSAID comparator. Unmarked studies use placebo or no drug comparator. Double asterisk study has both a non-selective NSAID and placebo comparator arms.

### Bleeding

#### Primary bleeding

A total of four studies^17^^,^^32^^,^^33^^,^^42^ included non-zero incidence data on primary bleeding (occurring within 24 hours of surgery).

**Figure 2.**
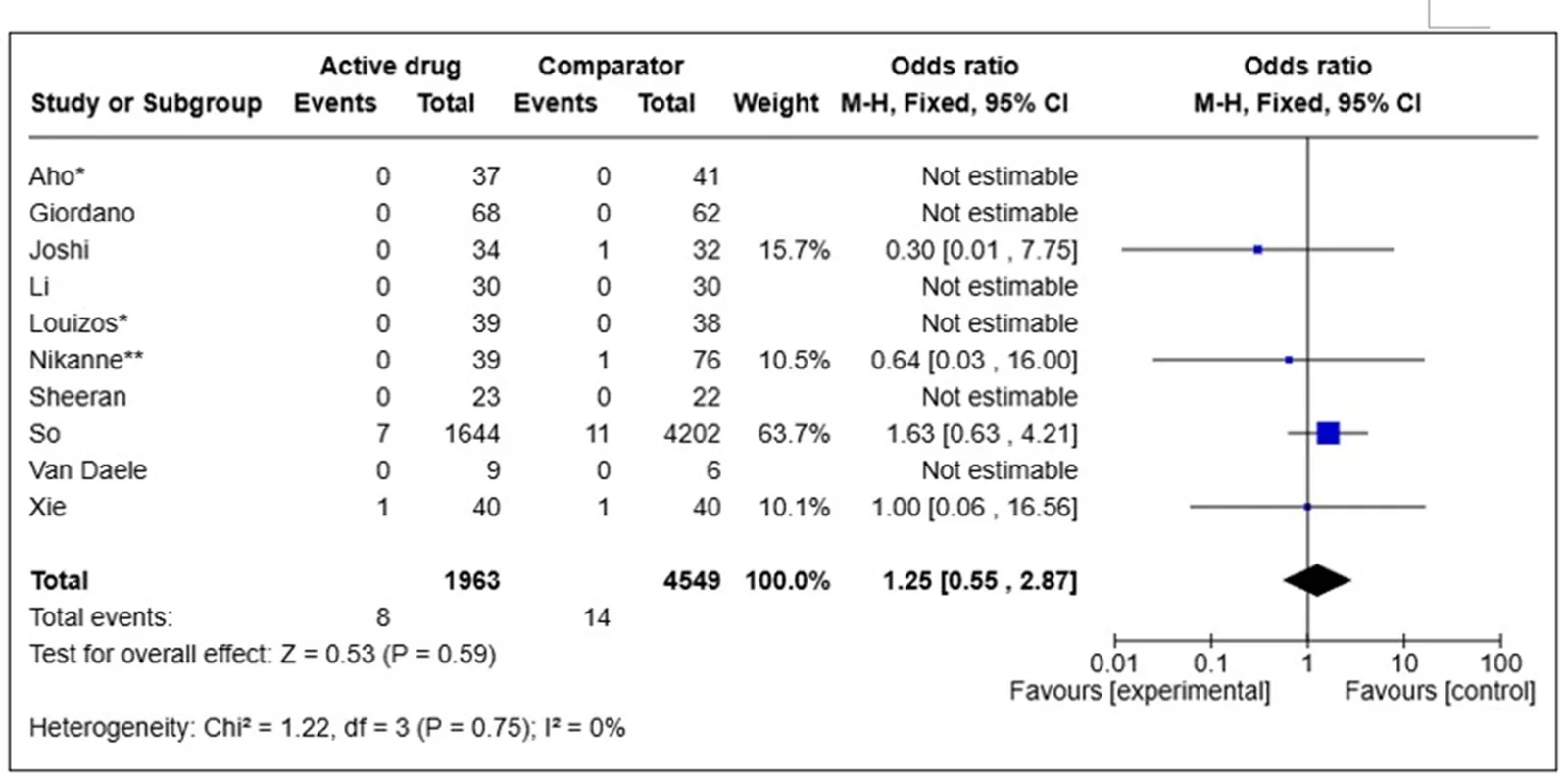
Mantel-Haenszel summary risk difference for incidence of primary bleeding: fixed effects model. Figure 2 long description.

Using the fixed-effect Mantel–Haenszel model, the pooled analysis demonstrated no significant difference in the incidence of bleeding between selective COX-2 inhibitors and all comparators (Z = 0.53; *p* = 0.59) (Figure 2). There was no evidence of statistical heterogeneity (I^2^ = 0 per cent; *p* = 0.75). However, given that only four studies were included, the power to detect true heterogeneity is limited.

Given the high proportion of zero-event studies, a sensitivity analysis was conducted using the Peto odds ratio (OR) method, which is recommended for sparse binary data and rare events.^46^ The pooled estimate from this analysis was OR = 1.27 (95 per cent CI: 0.51 to 3.16; *p* = 0.61), consistent with the findings from the primary analysis.

#### Secondary bleeding

Four studies^35^^,^^37^^,^^42^^,^^43^ clearly differentiated primary (occurring within 24 hours of surgery) and secondary bleeding (occurring more than 24 hours after surgery) and provided a minimum follow-up period of two weeks. Only two of these studies^35^^,^^42^ had non-zero incidence of secondary bleeding, so meaningful meta-analysis was unable to be performed. There was no significant difference in secondary bleeding within either study (Table 3).

**Table 3.** Summary of clinical outcomes Table 3 long description.

	Bleeding				Efficacy			Adverse effects		
Study	Primary	Secondary	Overall	Transfusion	Readmission for analgesia	Average number of doses of PRN analgesia	Number of patients needing PRN analgesia	PONV	GI bleeding	Abdominal pain
Ng, 2017		C^		C	D	D^		D*		
Xie, 2012	=						=^	=		
Joshi, 2003	C					C		C		
Van Daele, 2016	=	=	=		=	C^	C	C		
Murto, 2015			D^			C*^		=		D
So, 2024	D	D	D	C						
Giordano, 2023	=	=	=			C		D		D
Nikanne, 2005	C			=		C^				
Sheeran, 2016	=					=^		D		
Louizos, 2006	=					D^	C*	C	=	=
Aho, 2003	=	C	C	=		=	D	C		C
Naesh, 2005						C^		C		
Li, 2016	=					=^	C*	C*		
Lee, 2004		C^	C^	C^			D	C		C
Lestiansky, 2011	C^					C*^	C			

*Note:* Legend: C (green) result favours control (higher incidence). D (red) result favours study drug (higher incidence). = (orange) highly similar result. ^unable to include in meta-analysis due to non-equivalent data. *statistically significant result within the single study.

#### Overall bleeding

Overall bleeding, also defined as post-operative bleeding at any time requiring re-operation, was captured in studies with a minimum follow-up of two weeks and did not require differentiation between primary and secondary bleeding events. Four studies^35^^,^^37^^,^^42^^,^^43^ reported data on overall bleeding, of which only two studies^35^^,^^42^ had at least one event in either group. There was no significant difference in overall bleeding in either study (Table 3).

#### Number of patients requiring transfusion for bleeding

Five studies^17^^,^^30^^,^^34^^,^^35^^,^^42^ reported the number of patients requiring blood transfusion for bleeding. In the one study^42^ with analysable data meeting inclusion criteria, there was no significant difference in transfusion requirement (Table 3).

## Secondary outcomes

### Efficacy

#### Average number of doses of PRN analgesia

**Figure 3.**
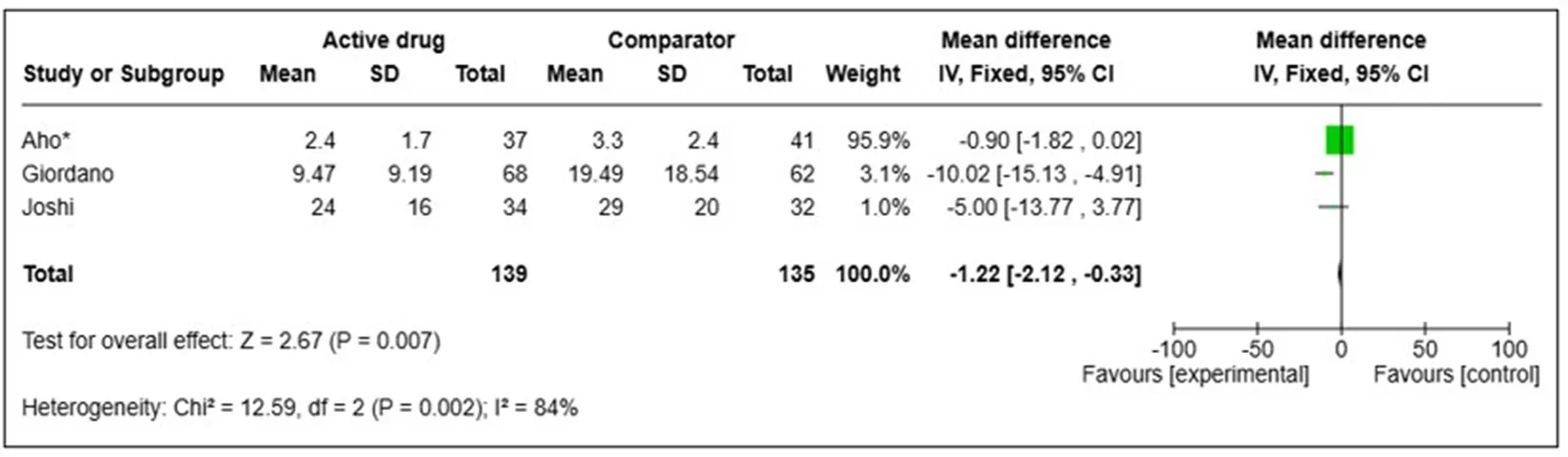
Forest plot using inverse variance for average number of doses of PRN analgesia: fixed effects model. Figure 3 long description.

Twelve studies reported the number of doses of PRN analgesia for patients; however, only three studies^32^^,^^35^^,^^37^ reported this outcome in a manner which was comparable between studies (the remainder either did not include a standard deviation, reported only median values or used aliquot-style titration of intravenous analgesia). Using the Mantel–Haenszel fixed-effect model, patients treated with selective COX-2 inhibitors required fewer doses of PRN analgesia (pooled mean difference was -1.22 (95 per cent CI, -2.12 to -0.33; Z = 2.67; *p* = 0.007; I^2^ = 84 per cent) [Figure 3]) with substantial heterogeneity. The weakest effect was observed in the only study^35^ which compared a selective COX-2 inhibitor with an active non-selective NSAID comparator drug (ibuprofen) rather than placebo.

#### Number of patients needing PRN analgesia

Six studies^30^^,^^31^^,^^33^^,^^39^^,^^41^^,^^43^ reported the number of patients requiring PRN analgesia: four^31^^,^^33^^,^^41^^,^^43^ using placebo comparator and two^30^^,^^39^ using an NSAID comparator. Pooled results showed no significant difference in the number of patients needing PRN analgesia in analysis that included all comparators. As expected, heterogeneity was high when considering all comparators (I^2^ = 78 per cent; *p* = 0.0004). Lee *et al*.^30^ was the only study to consider post-discharge administration of PRN analgesia, the remaining five studies assessed peri-operative administration of PRN analgesia.

The subgroup analysis of the four studies^31^^,^^33^^,^^41^^,^^43^ with placebo comparator only showed that significantly fewer patients needed PRN analgesia when treated with selective COX-2 inhibitors compared to placebo (OR 0.19, 95 per cent CI, 0.08-0.47; Z = 3.58; *p* = 0.0003 fixed effects model), (OR 0.23, 95 per cent CI, 0.09-0.59; Z = 3.04; *p* = 0.002 random effects model). Heterogeneity was low. Each of these studies reported on peri-operative administration of PRN analgesia rather than post-discharge.

#### Number of readmissions for pain

Only two studies^34^^,^^47^ reported the number of patients readmitted for pain needing analgesia with no significant effect of selective COX-2 inhibitors in either individual study.

### Adverse effects

#### Post-operative nausea and vomiting

**Figure 4.**
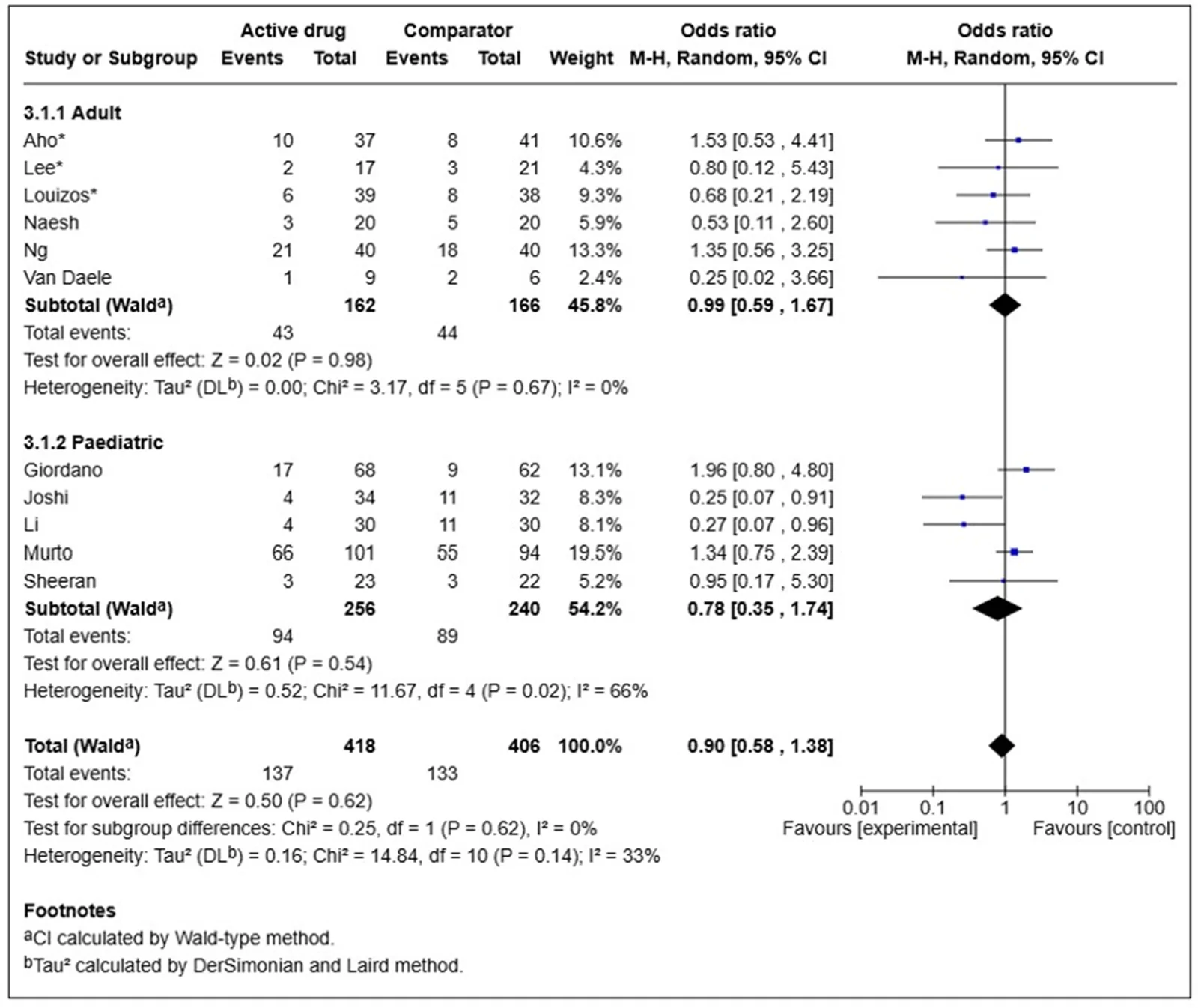
Forest plot Mantel-Haenszel summary risk difference for incidence of post operative nausea and vomiting: random effects model. Figure 4 long description.

Eleven studies (six adult and five paediatric) reported incidence of post-operative nausea and vomiting (Figure 4). There was no significant difference in the incidence of post-operative nausea and vomiting (PONV) between selective COX-2 inhibitors and comparators, regardless of fixed or random effects model, or in subgroup analysis between adult and paediatric patients. Three studies (all in adult patients) used non-selective NSAID comparators.

Analysis was also conducted for comparator groups separately. There was no significant difference in PONV with placebo or NSAID comparators.

**Figure 5.**
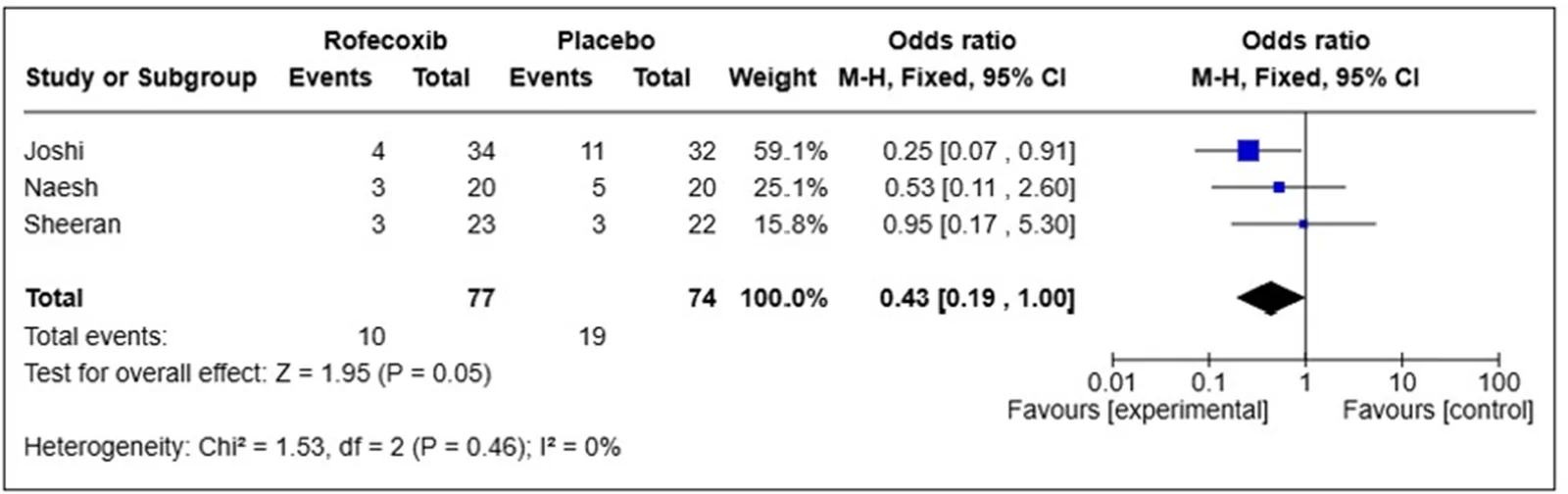
Forest plot Mantel-Haenszel summary risk difference for incidence of post operative nausea and vomiting with rofecoxib only: fixed effect model. Figure 5 long description.

**Figure 6.**
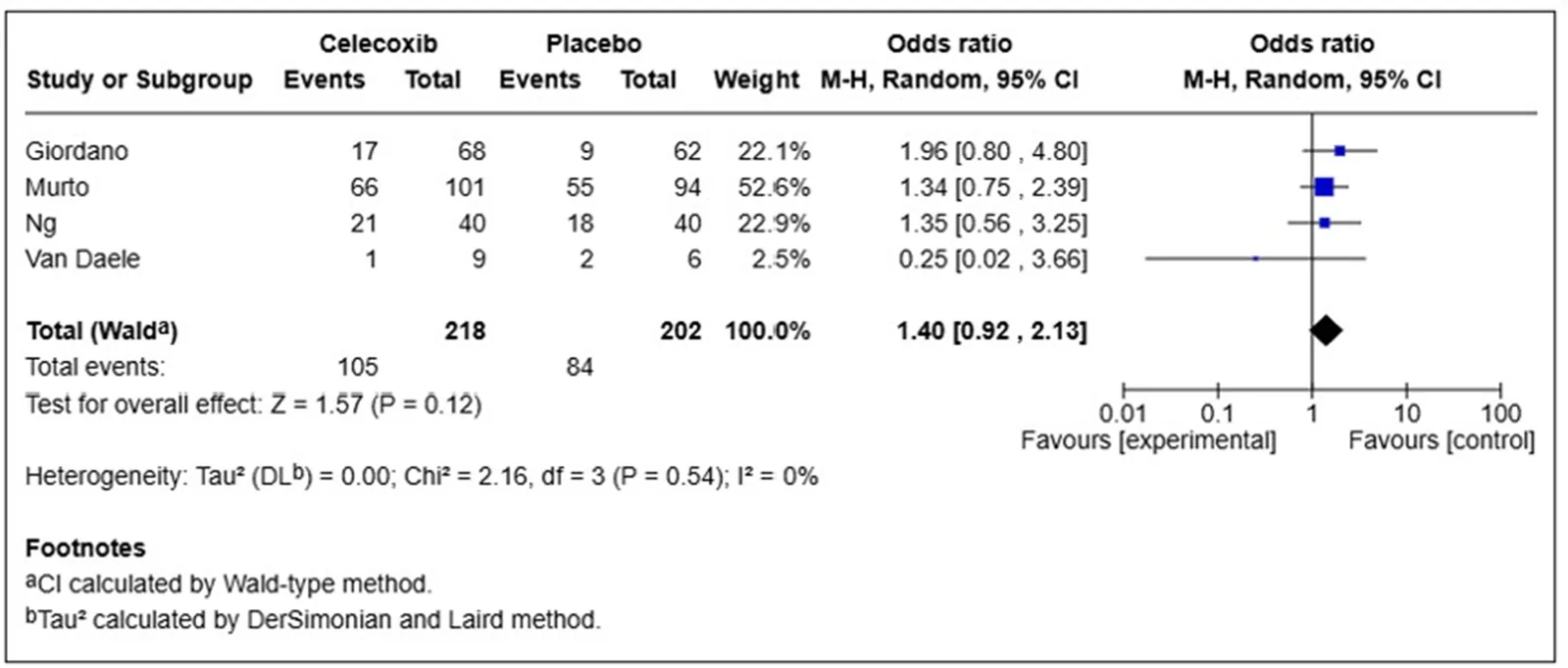
Forest plot Mantel-Haenszel summary risk difference for incidence of post operative nausea and vomiting with celecoxib only: random effects model. Figure 6 long description.

There were sufficient studies comparing celecoxib and rofecoxib with placebo to conduct meta-analysis of the effect of both drugs on PONV separately (Figures 5, 6).

Using a fixed-effect model, pooling three studies comparing rofecoxib with placebo suggested a reduction in odds; however, the effect was borderline, with the 95 per cent CI including the null value (OR = 0.43; 95 per cent CI, 0.19-1.00; Z = 1.95; *p* = 0.05). Heterogeneity was low.

There was no significant difference in the incidence of PONV with celecoxib compared to placebo among 4 studies (Figure 6). Only one study^43^ reported a decreased odds ratio of PONV with celecoxib; this study had the smallest sample size. Sensitivity analysis excluding this study did not materially change the results.

#### Abdominal pain

Five studies^30^^,^^35^^–^^37^^,^^39^ reported incidence of abdominal pain, three of which used non-selective NSAIDs as comparators (the other two using placebo comparators). There was no significant difference in abdominal pain between selective COX-2 inhibitors and comparators. One study^39^ had zero incidence of abdominal pain in both groups and was unable to be included in meta-analysis.

#### Other adverse effects

Only one study^39^ reported on the incidence of gastrointestinal bleeding with selective COX-2 inhibitors, and no events occurred in either group. None of the included studies reported the incidence of acute kidney injury or cardiac events.

#### Risk of bias

The GRADE^48^ summary indicated high certainty of evidence that selective COX-2 inhibitors do not increase risk of overall bleeding, reduce the need for PRN analgesia compared to all comparators, and do not increase PONV. There was also high certainty that rofecoxib does not increase PONV. The remaining outcomes were downgraded primarily for imprecision due to weak power calculations and substantial contribution from studies with a high risk of bias (Appendix D).

Power for bleeding outcomes was calculated using noninferiority limits aligned with a previously published Cochrane review^18^ on post-tonsillectomy bleeding risk with NSAIDs (d = 2.5 per cent; significance level 2.5 per cent, two-tailed in this review given the combined analysis of both active and placebo comparators). For analgesic efficacy and side effect outcomes power calculations used a 5 per cent significance level: two-tailed for combined comparators and one-tailed for placebo comparators, with a non-inferiority limit of 10 per cent where appropriate.

## Discussion

### Primary outcomes: bleeding

The current systematic review found no evidence of an increased bleeding risk associated with selective COX-2 inhibitors across all primary outcome measures, including primary, secondary, overall bleeding and transfusion.

Confidence in the certainty of evidence varied across outcomes, as reflected in the GRADE assessments. This was largely attributable to limitations in outcome reporting and comparator separation, particularly where active comparators and placebo or no-drug controls could not be fully distinguished. Although bleeding was commonly reported, it was frequently measured using non-standardised or surrogate indicators (for example, the number of intraoperative swabs used) as it is hard to measure the amount of bleeding in a patient presenting to the emergency department. In addition, the incidence of severe bleeding was low, and many studies identified in this systematic review did not contribute to meta-analysis due to zero events in both treatment groups. While these factors limit the ability to determine whether selective COX-2 inhibitors are superior to ibuprofen or other commonly used non-selective NSAIDs in terms of bleeding risk, the review strengthen confidence that selective COX-2 inhibitors do not confer a direct increase in bleeding risk. Given that safety was demonstrated when selective COX-2 inhibitors were compared with both placebo and active non-selective NSAID comparators combined, it is reasonable to infer safety relative to placebo alone.

Comparison with previous systematic reviews provides important contextualisation of these findings. Earlier reviews suggested an increased risk of post-tonsillectomy bleeding with NSAIDs^3^^–^^5^; however, more recent systematic reviews published within the past five years consistently report no increased bleeding risk. Banatwala^25^ found no increased bleeding risk with celecoxib compared to placebo, while Teerawattanon^49^ examined selective COX-2 inhibitors across multiple surgical procedures, including tonsillectomy, and similarly found no increased bleeding risk, even in subgroup analyses. The consistency of findings across NSAID classes and surgical contexts provides additional reassurance regarding the bleeding safety of selective COX-2 inhibitors in tonsillectomy patients.

Finally, it is important to acknowledge that different bleeding outcomes are subject to distinct sources of bias. Primary bleeding is more likely to be influenced by surgical haemostasis, whereas secondary bleeding is more closely associated with other factors, such as poor oral intake or inadequate analgesia.^50^ Transfusion, as a marker of bleeding severity, is particularly vulnerable to reporting bias due to its rarity (<1 per cent)^11^ and the lack of standardised transfusion thresholds across studies.

### Secondary outcomes

#### Number of patients needing PRN analgesia

Measures of analgesic efficacy were predictably influenced by the choice of comparator. When all comparators were considered together, there was no significant difference in the number of patients requiring PRN analgesia between selective COX-2 inhibitors and comparator groups. However, when analyses were restricted to placebo-controlled studies, selective COX-2 inhibitors were associated with a significant reduction in the proportion of patients requiring PRN analgesia.

The two studies^14^^,^^15^ demonstrating inferior analgesic efficacy—reflected by a higher proportion of patients requiring PRN analgesia—used active NSAID comparators (ketoprofen and ibuprofen).

In contrast, when selective COX-2 inhibitors were compared with placebo alone, the proportion of patients requiring peri-operative PRN analgesia was nearly five-fold lower. This large and consistent effect provides a high degree of confidence in analgesic efficacy and is more encouraging than findings from a 2018 systematic review,^18^ which concluded that the efficacy of selective COX-2 inhibitors in paediatric tonsillectomy patients remained unclear. Importantly, three of the four studies included in the placebo-only meta-analysis were conducted in adult populations, which may partially explain the clearer signal of benefit observed in this review.

#### Average number of doses of PRN analgesia

Fewer PRN analgesia doses were required in patients treated with selective COX-2 inhibitors compared with both placebo and non-selective NSAIDs. Aho^35^ used ibuprofen as the active comparator and found similar analgesic efficacy between ibuprofen and nimesulide; notably, this was the only study conducted in an adult population. In this study, ibuprofen was administered at a low dose (200 mg per dose), and outcome data were drawn from both peri-operative and post-discharge settings. In contrast, Giordano^37^ administered a higher dose of celecoxib (12 mg/kg) than that used in most other included studies (typically 6 mg/kg), which may have influenced observed efficacy.

Statistical significance was observed only when a fixed-effect model was applied. Whilst this approach may be appropriate given the small number of included studies, heterogeneity remained high, likely reflecting variation in comparators, dosing regimens, and clinical settings.

Despite this variability, there is reasonable confidence that selective COX-2 inhibitors provide clinically meaningful analgesia following tonsillectomy. Celecoxib demonstrated equivalent efficacy to low-dose ibuprofen in one study; however, it remains unclear whether celecoxib is inferior to standard adult dosing of ibuprofen (400 mg per dose) or to other non-selective NSAIDs. This uncertainty is consistent with findings from an earlier systematic review by Chen^51^, which assessed selective COX-2 inhibitors for post-operative dental pain. In that review, rofecoxib and valdecoxib were more effective than oxycodone/paracetamol and similarly effective to 400 mg ibuprofen, whereas 200 mg celecoxib was less effective than 400 mg ibuprofen. When adult dosing is considered, a 200 mg dose of celecoxib approximates a 12 mg/kg paediatric dose, acknowledging differences in drug metabolism between children and adults.^21^ Taken together, these findings suggest that higher doses of celecoxib may achieve analgesic efficacy comparable to low-dose ibuprofen, but may provide inferior analgesia compared with maximally safe doses of either drug.

#### PONV

The available evidence suggests that selective COX-2 inhibitors do not increase the incidence of PONV. PONV was consistently reported across included studies and was well suited to meta-analysis, given its relative ease of quantification and standardisation using patient- or carer-reported questionnaires and the use of antiemetic medications. However, peri-operative PONV measurement methods were not routinely described, which may have contributed to some variability across studies.

The meta-analysis was adequately powered to demonstrate non-inferiority when selective COX-2 inhibitors were compared with placebo. In contrast, analyses assessing superiority were underpowered, with an estimated statistical power of 0.53, limiting the ability to detect clinically meaningful differences. When examined individually, celecoxib compared with placebo was more likely to be inferior with respect to PONV, although analyses were not sufficiently powered to demonstrate statistical significance. Rofecoxib, by contrast, was not inferior to placebo, with an adequate sample size to support this conclusion. A small superiority margin was present but remained underpowered (power = 0.52). If extrapolated to larger sample sizes, these opposing drug-specific effects—higher PONV with celecoxib and lower PONV with rofecoxib—may partially explain the overall non-inferiority finding observed in pooled analyses.

Pharmacological differences between selective COX-2 inhibitors may provide a biological explanation for these findings. Rofecoxib has a much higher IC80 than celecoxib (> 200 compared to 9),^14^ and the decreased COX-1 affinity for gastric mucosa would be a reasonable explanation for reduced PONV with rofecoxib vs placebo compared to celecoxib vs placebo. Additionally, the variable efficacy of celecoxib as discussed above may potentiate PONV with increased use of opiate medication to obtain adequate analgesia (nausea is a known opiate side effect).^52^ The observed reduction in PONV with selective COX-2 inhibitors compared with placebo is most plausibly explained by opioid-sparing effects resulting from effective regular analgesia.

Comparison with previous literature provides additional context. A previous systematic review by Straube *et al*.^53^ examining pre-operative selective COX-2 inhibitor use across surgical procedures (including rofecoxib, parecoxib and celecoxib) found no individual trial or pooled analysis demonstrating an increased incidence of PONV.

### Limitations

Despite rigorous methodology, this systematic review is subject to several inherent limitations. The decision to pool multiple selective COX-2 inhibitors and comparator types was necessary to achieve meaningful statistical power but constrained the ability to conduct subgroup analyses and explore heterogeneity in a robust manner. As a result, potential individual drug-specific effects may have been obscured.

The systematic review process is also influenced by the construction of the search strategy, which relies on clinical knowledge and experience to capture all relevant terminology. Although this risk was mitigated through iterative search refinement, incomplete retrieval of relevant studies remains possible. Although database searches could identify non-English publications, indexing of titles and abstracts in English may limit retrieval of studies published in other languages. Additionally, studies are more likely to be conducted, published, and cited in high-income settings, potentially introducing geographic and socioeconomic publication bias.^39^

Overall, the certainty of evidence was rated as moderate to high for most outcomes. Downgrading typically occurred due to imprecision, particularly in subgroup analyses, or due to heterogeneity arising from the inclusion of multiple drugs and comparators. Importantly, the methodological quality of individual studies was generally good.

### Implications for clinical practice

NSAIDs are well-established as safe and effective for use in tonsillectomy patients. This review reinforces that position, showing that selective COX-2 inhibitors are a safe alternative in terms of bleeding risk while providing clinically meaningful analgesia. The opioid-sparing effects of selective COX-2 inhibitors may contribute to a reduction in gastrointestinal adverse effects, such as nausea and vomiting, although these effects appear to vary between agents.

Although selective COX-2 inhibitors have been shown in vitro to inhibit platelet aggregation less than non-selective NSAIDs, these pharmacological differences do not translate into clinically measurable reductions in bleeding risk following tonsillectomy. Historically conflicting results likely reflect the multifactorial nature of post-tonsillectomy bleeding, the low incidence of severe bleeding events and patient heterogeneity across studies.

Clinical applicability must also consider regulatory, practical, and economic constraints. Rofecoxib was withdrawn from international markets over twenty years ago and is not relevant to current clinical practice. Evidence from this review suggests that greater COX-2 selectivity may be associated with reduced gastrointestinal adverse effects, but potentially higher cardiovascular risk—an important consideration for adult patients with comorbidities.

In Australia, celecoxib and etoricoxib are not TGA-approved for patients under 18 years of age, rendering their paediatric use off-label. Parecoxib, administered intravenously and typically as a single peri-operative dose, is TGA-approved across age groups and provides a practical option in peri-operative analgesia, although it is less accessible and more costly than oral alternatives. Celecoxib costs approximately AUD $23 for a two-week course, whereas etoricoxib exceeds AUD $60 for the same duration, depending on the retailer. By contrast, non-selective NSAIDs such as ibuprofen remain widely available and cost-effective (less than $6 for 2 weeks), making them the most practical first-line option for most patients.

Selective COX-2 inhibitors represent a safe alternative when gastrointestinal tolerability or opioid-sparing effects are priorities. However, given the lack of demonstrated superiority over non-selective NSAIDs, higher cost, regulatory restrictions, and uncertainties regarding drug-specific efficacy and cardiovascular risk, non-selective NSAIDs remain an appropriate first-line choice. Selective COX-2 inhibitors may be considered on a case-by-case basis, particularly for patients with higher risk of gastrointestinal adverse effects or where minimising opioid use is a clinical objective.

### Suggested clinical considerations based on review findings

Based on the evidence from this systematic review and the broader literature on selective COX-2 inhibitors, patient considerations that may be relevant when selecting post-operative analgesia for tonsillectomy have been summarised in Table 4.

**Table 4.** Indications for treatment with selective COX-2 inhibitors or non-selective NSAIDs in tonsillectomy patients Table 4 long description.

Selective COX−2 inhibitors	Non-selective NSAIDS
Patients without a history of ischaemic heart disease or stroke. Patients for whom reduced dosing frequency is desirable to increase adherence (e.g., twice daily instead of three times daily). Patients with a history of peptic ulcer disease who have inadequate analgesia with paracetamol and opioids alone. Patients who have discontinued non-selective NSAIDs due to abdominal pain. Patients vulnerable to reduced oral intake following surgery (e.g., young children, individuals with intellectual disability or patients with poor appetite at baseline).	Patients for whom additional medication costs are prohibitive. Patients who experience inadequate analgesia with celecoxib treatment.

These considerations are *interpretive guidance* informed by pharmacological properties, regulatory approvals and clinical context, rather than direct findings of the systematic review. Clinicians should weigh individual patient factors, cost and regulatory restrictions when choosing an appropriate analgesic regimen.

### Implications for research

A key limitation in this field is the lack of standardised, comparable outcome measures, which prevented the inclusion of many data points in meta-analysis. Variability in reporting methods limited the ability to synthesise evidence across studies. Future research would benefit from the adoption of standardised, universally applicable outcome measures. The Stammberger grading system^54^ has been previously used to report post-tonsillectomy bleeding. It captures mild to serious bleeding events and classifies them according to severity (including distinct categories for reoperation and transfusion) along with the number of days post operation the bleed occurs. Researchers can consider using this grading system as a systematic way to report both the incidence and severity of post-tonsillectomy bleeding.

Although many studies have focused on peri-operative or inpatient data collection, post-tonsillectomy pain peaks (and largest cumulative analgesic requirement) often occur after discharge. Consequently, future studies assessing the safety and efficacy of selective COX-2 inhibitors would be more informative if conducted in outpatient or home-based settings, capturing real-world analgesic use and adverse events. Combining self-reported outcomes with more objective measures such as readmissions to hospital or the number of doses of a PRN medication can provide a more complete picture of analgesic efficacy and adverse effects.

The emerging role of selective COX-2 inhibitors is likely to be most relevant for patients at increased baseline risk of gastrointestinal side effects. To determine the optimal patient populations and dosing strategies, more high-quality, standardised and adequately powered studies are required. Future research should aim to balance rigorous methodological design with real-world applicability, including post-discharge follow-up and patient-centred outcomes, to guide safe and effective analgesic choices in tonsillectomy care.

**Summary**

- The use of NSAIDs in tonsillectomy patients can be controversial due to bleeding concerns
- Selective COX-2 inhibitors should be safer than non-selective NSAIDs in minimising bleeding risk
- There is no increased risk of bleeding in tonsillectomy patients with selective COX-2 inhibitor use
- Superiority to non-selective NSAIDS has not been demonstrated with regards to bleeding risk
- Some selective COX-2 inhibitors may be less effective than ibuprofen, but this needs further drug-specific research
- Selective COX-2 inhibitor use may reduce nausea and vomiting by limiting opioid use post-operatively

## Conclusion

This systematic review demonstrates that selective COX-2 inhibitors are safe from a bleeding perspective and provide effective analgesia compared with placebo in patients undergoing tonsillectomy. Across primary, secondary and overall bleeding outcomes, selective COX-2 inhibitors were not associated with increased risk compared with either placebo or non-selective NSAIDs. Evidence trends suggest variability between individual selective COX-2 inhibitors. Some agents may provide less effective analgesia than non-selective NSAIDs, while more highly COX-2–selective agents may reduce gastrointestinal adverse effects, including post-operative nausea and vomiting. However, these observations are based on limited and heterogeneous data, and further research is required to establish certainty regarding drug-specific efficacy and safety profiles.

## Supporting information

10.1017/S0022215126105039.sm001 Dunmall et al. supplementary material Dunmall et al. supplementary material

## Supplementary material

The supplementary material for this article can be found at https://doi.org/10.1017/S0022215126105039.

## Table 1 Long description

The flowchart outlines the process of study selection in three main phases: Identification, Screening and Included. In the Identification phase, records are gathered from databases and registers totaling 1089 and from other sources totaling 5. Records removed amount to 520 due to duplicates and other reasons. In the Screening phase, 574 records are screened, with 538 excluded. Records sought for retrieval are 36, with 13 not retrieved. Records assessed for eligibility are 23, with 8 studies excluded for various reasons. In the Included phase, 15 records are included in the final review.

Navigate back to Table 1.

## Figure 2 Long description

Active drug Comparator Study or Subgroup Events Total Events Total Weight Odds ratio M-H, Fixed, 95 percent CI Aho asterisk 0 37 0 41 Not estimable Giordano 0 68 0 62 Not estimable Joshi 0 34 1 32 15.7 percent 0.30 (0.01, 7.75) Li 0 30 0 30 Not estimable Louizos asterisk 0 39 0 38 Not estimable Nikkane asterisk 0 39 1 76 10.5 percent 0.64 (0.03, 16.00) Sheeran 0 23 0 22 Not estimable So 7 1644 11 4202 63.7 percent 1.63 (0.63, 4.21) Van Daele 0 9 0 6 Not estimable Xie 1 40 1 40 10.1 percent 1.00 (0.06, 16.56) Total 1963 4549 100.0 percent 1.25 (0.55, 2.87) Total events: 8 14 Test for overall effect: Z equals 0.53 (P equals 0.59) Heterogeneity: Chi squared equals 1.22, df equals 3 (P equals 0.75); I squared equals 0 percent Odds ratio M-H, Fixed, 95 percent CI A horizontal axis is labeled 0.01, 0.1, 1, 10, 100. Text under the axis reads: Favours (experimental) on the left and Favours (control) on the right. Plotted study estimates shown as squares with horizontal confidence intervals: Joshi: odds ratio 0.30 with confidence interval 0.01 to 7.75. Nikkane asterisk: odds ratio 0.64 with confidence interval 0.03 to 16.00. So: odds ratio 1.63 with confidence interval 0.63 to 4.21. Xie: odds ratio 1.00 with confidence interval 0.06 to 16.56. The pooled estimate is shown as a diamond at 1.25 with confidence interval 0.55 to 2.87.

Navigate back to Figure 2.

## Figure 3 Long description

Study or Subgroup A forest plot with a table on the left and an effect-size axis on the right. Table columns are: Study or Subgroup; Active drug with subcolumns Mean, SD, Total; Comparator with subcolumns Mean, SD, Total; Weight; Mean difference IV, Fixed, 95 percent CI. Row 1: Aho. Active drug Mean 2.4, SD 1.7, Total 37. Comparator Mean 3.3, SD 2.4, Total 41. Weight 95.9 percent. Mean difference minus 0.90 with 95 percent CI minus 1.82 to 0.02. Row 2: Giordano. Active drug Mean 9.47, SD 9.19, Total 68. Comparator Mean 19.49, SD 18.54, Total 62. Weight 3.1 percent. Mean difference minus 10.02 with 95 percent CI minus 15.13 to minus 4.91. Row 3: Joshi. Active drug Mean 24, SD 16, Total 34. Comparator Mean 29, SD 20, Total 32. Weight 1.0 percent. Mean difference minus 5.00 with 95 percent CI minus 13.77 to 3.77. Total row: Active drug Total 139. Comparator Total 135. Weight 100.0 percent. Mean difference minus 1.22 with 95 percent CI minus 2.12 to minus 0.33. Text below the table: Test for overall effect: Z equals 2.67 (P equals 0.007). Heterogeneity: Chi squared equals 12.59, df equals 2 (P equals 0.002); I squared equals 84 percent. Right-side axis label: Mean difference IV, Fixed, 95 percent CI. The axis has tick labels at minus 100, minus 50, 0, 50, 100. A vertical reference line is drawn at 0. Plotted study markers: Aho is plotted at minus 0.90 with a horizontal line from minus 1.82 to 0.02. Giordano is plotted at minus 10.02 with a horizontal line from minus 15.13 to minus 4.91. Joshi is plotted at minus 5.00 with a horizontal line from minus 13.77 to 3.77. Pooled effect marker: a diamond centered at minus 1.22 with left and right points at minus 2.12 and minus 0.33. Bottom labels: Favours [experimental] on the left and Favours [control] on the right.

Navigate back to Figure 3.

## Figure 4 Long description

Active drug Comparator Study or Subgroup Events Total Events Total Weight M-H, Random, 95 percent CI 3.1.1 Adult Aho* 10 37 8 41 10.6 percent 1.53 [0.53, 4.41] Lee* 2 17 3 21 4.3 percent 0.80 [0.12, 5.43] Louizos* 6 39 8 38 9.3 percent 0.68 [0.21, 2.19] Naesh* 3 20 5 20 5.9 percent 0.53 [0.11, 2.60] Ng 21 40 18 40 13.3 percent 1.35 [0.56, 3.25] Van Daele 1 9 2 6 2.4 percent 0.25 [0.02, 3.66] Subtotal (Wald*) 43 162 44 166 45.8 percent 0.90 [0.59, 1.67] Total events: 43 44 Test for overall effect: Z = 0.02 (P = 0.98) Heterogeneity: Tau squared = 0.00; Chi squared = 3.17, df = 5 (P = 0.67); I squared = 0 percent 3.1.2 Paediatric Giordano 17 68 9 62 13.1 percent 1.96 [0.80, 4.80] Joshi 4 34 11 32 8.3 percent 0.25 [0.07, 0.91] Li 4 30 11 30 8.1 percent 0.27 [0.07, 0.96] Murto 66 101 55 94 19.5 percent 1.34 [0.75, 2.39] Shearer 3 23 3 22 5.2 percent 0.95 [0.17, 5.30] Subtotal (Wald*) 94 256 89 240 54.2 percent 0.78 [0.35, 1.74] Total events: 94 89 Test for overall effect: Z = 0.54 (P = 0.59) Heterogeneity: Tau squared = 0.52; Chi squared = 11.67, df = 4 (P = 0.02); I squared = 66 percent Total (Wald*) 137 418 133 406 100.0 percent 0.90 [0.58, 1.38] Total events: 137 133 Test for overall effect: Z = 0.50 (P = 0.62) Test for subgroup differences: Chi squared = 0.25, df = 1 (P = 0.62), I squared = 0 percent Heterogeneity: Tau squared = 0.16; Chi squared = 14.84, df = 10 (P = 0.14); I squared = 33 percent Odds ratio M-H, Random, 95 percent CI Scale labels: 0.01, 0.1, 1, 10, 100 Axis captions: Favours [experimental] on the left and Favours [control] on the right. Footnotes CI calculated by Wald-type method. Tau squared calculated by DerSimonian and Laird method.

Navigate back to Figure 4.

## Figure 5 Long description

Study or Subgroup table with columns labeled Rofecoxib, Placebo, Weight and Odds ratio M-H, Fixed, 95 percent CI. Row: Joshi. Rofecoxib Events 1, Total 34. Placebo Events 11, Total 32. Weight 59.1 percent. Odds ratio 0.25 [0.07, 0.91]. Row: Naesh. Rofecoxib Events 3, Total 20. Placebo Events 5, Total 20. Weight 25.1 percent. Odds ratio 0.53 [0.11, 2.60]. Row: Sheeran. Rofecoxib Events 3, Total 23. Placebo Events 3, Total 22. Weight 15.8 percent. Odds ratio 0.95 [0.17, 5.30]. Row: Total. Rofecoxib Total 77. Placebo Total 74. Weight 100.0 percent. Odds ratio 0.43 [0.19, 1.00]. Text below the table: Total events: 10 and 19. Test for overall effect: Z equals 1.96 (P equals 0.05). Heterogeneity: Chi squared equals 1.53, df equals 2 (P equals 0.46); I squared equals 0 percent. Forest plot at right with x-axis labeled Odds ratio on a logarithmic scale with tick labels 0.01, 0.1, 1, 10, 100. A vertical reference line is drawn at 1. Three square markers with horizontal confidence intervals align with the three study rows and a diamond marker aligns with the Total row. Bottom labels read Favours [experimental] on the left and Favours [control] on the right.

Navigate back to Figure 5.

## Figure 6 Long description

Celecoxib Placebo A forest plot with a left table and a right odds ratio plot. The table columns are labeled: Study or Subgroup; Celecoxib with subcolumns Events and Total; Placebo with subcolumns Events and Total; Weight; Odds ratio; M-H, Random, 95 percent CI. Study rows: Giordano: Celecoxib Events 17, Total 68; Placebo Events 9, Total 62; Weight 22.1 percent; Odds ratio 1.96 [0.80, 4.80]. Murto: Celecoxib Events 66, Total 101; Placebo Events 55, Total 94; Weight 52.6 percent; Odds ratio 1.34 [0.75, 2.39]. Ng: Celecoxib Events 21, Total 40; Placebo Events 18, Total 40; Weight 22.9 percent; Odds ratio 1.35 [0.56, 3.25]. Van Daele: Celecoxib Events 1, Total 9; Placebo Events 2, Total 6; Weight 2.5 percent; Odds ratio 0.25 [0.02, 3.66]. Total (Wald): Celecoxib Total 218; Placebo Total 202; Weight 100.0 percent; Odds ratio 1.40 [0.92, 2.13]. Total events: Celecoxib 105; Placebo 84. Test for overall effect: Z 1.57 (P 0.12). Heterogeneity: Tau squared 0.00; Chi squared 2.16, df 3 (P 0.54); I squared 0 percent. The right plot header reads: Odds ratio; M-H, Random, 95 percent CI. The horizontal axis is labeled with tick values 0.01, 0.1, 1, 10, 100. A vertical reference line is drawn at 1. Each study is shown as a square with a horizontal confidence interval line at the listed odds ratio and confidence interval. The pooled estimate is shown as a diamond at 1.40 with endpoints 0.92 and 2.13. Text below the axis reads: Favours [experimental] on the left and Favours [control] on the right. Footnotes: aCI calculated by Wald-type method. bTau squared calculated by DerSimonian and Laird method.

Navigate back to Figure 6.

## Table 2 Long description

The table compares 15 studies by care setting, indication, surgeon experience, surgical method, drug route, daily dose, and total dose. Most studies used oral dosing, while intravenous dosing appears in two peri-operative studies and one study used oral or intramuscular dosing. Settings vary: several are peri-operative, several are post-discharge, and two span both peri-operative and post-discharge. Indications are most often recurrent infection, sometimes obstruction, and many entries do not state an indication. Surgical methods are frequently electrocautery or electrocautery dissection, with cold steel reported in a smaller number of studies and several methods not stated. Daily dosing ranges from 1 milligram per kilogram up to 12 milligrams per kilogram, or fixed doses from 50 milligrams to 400 milligrams; total dosing ranges from single-dose totals to extended totals such as 2800 milligrams and 4600 milligrams, with one study noting additional as-needed doses. Surgeon experience is inconsistently reported, with several studies listing an experienced surgeon and many not stating experience. Comparisons across studies are limited because dosing units mix fixed milligrams and weight-based milligrams per kilogram, and multiple fields are missing in several rows.

Navigate back to Table 2.

## Table 3 Long description

The table summarizes, by study, whether outcomes favored the control group, the study drug, or were highly similar across bleeding, efficacy, and adverse-effect measures. Bleeding is split into primary and secondary bleeding plus an overall bleeding measure, and efficacy includes transfusion, readmission for analgesia, and two measures of as-needed analgesic use. Adverse effects include postoperative nausea and vomiting, gastrointestinal bleeding, and abdominal pain. Findings vary by study and outcome: some studies report bleeding outcomes favoring control, while others report highly similar bleeding results or outcomes favoring the study drug. For efficacy, several studies indicate the study drug is favored for readmission for analgesia and for average as-needed analgesic dosing, but other studies favor control for analgesic-use measures. For adverse effects, postoperative nausea and vomiting often favors the study drug or is highly similar, though multiple studies favor control; abdominal pain is reported in fewer studies and is split between favoring control and favoring the study drug. Some entries are marked as statistically significant within a single study, and some outcomes could not be pooled in meta-analysis due to non-equivalent data, so comparisons should be interpreted cautiously.

Navigate back to Table 3.

## Table 4 Long description

The table lists patient situations that may guide choosing either a selective COX-2 inhibitor or a non-selective NSAID for pain control after tonsillectomy. Selective COX-2 inhibitors are indicated for patients without a history of ischaemic heart disease or stroke and for those who would benefit from reduced dosing frequency to improve adherence. They are also suggested for patients with a history of peptic ulcer disease who still have inadequate pain relief with paracetamol and opioids alone, and for patients who stopped non-selective NSAIDs due to abdominal pain. Another COX-2 group includes patients at risk of reduced oral intake after surgery, such as young children, people with intellectual disability, or those with poor baseline appetite. Non-selective NSAIDs are indicated when additional medication costs are prohibitive or when celecoxib does not provide adequate analgesia. The table provides indications rather than outcome data, so it does not quantify effectiveness or risk and should be applied alongside individual clinical assessment.

Navigate back to Table 4.
