# Supplementary material for: The safety of selective COX-2 inhibitors in tonsillectomy patients: a systematic review
Source: J Laryngol Otol. 2026 Aug;140(8):831–44. doi: 10.1017/S0022215126105039 (PMC13735496; doi:10.1017/S0022215126105039)
Supplement: Supplementary file 1 [file S0022215126105039sup001.zip › Appendix .docx]

Appendix A. MEDLINE (Ovid)

| Search | Terms | No. of records retrieved |
| --- | --- | --- |
| 1 | Tonsillectomy.sh or (tonsil* or otolaryngological or adenotonsil*).mp | 43340 |
| 2 | Exp cyclooxygenase inhibitors or (cyclo-oxygenase inhibitor* or NSAID* or cyclooxygenase inhibitor* or cyclo-oxygenase type 2 antagonist or cyclo-oxygenase type 2 inhibitor* or cyclooxygenase type 2 antagonist or cyclooxygenase type 2 inhibitor* or cyclooxygenase 2 inhibitor* or cyclo-oxygenase 2 inhibitor* or parecoxib or rofecoxib or celecoxib or COX-2).mp | 191619 |
| 3 | Exp hemorrhage or exp postoperative complications or exp pain or pain management.sh or pain measurement.sh or exp pain perception or (h?emorrhag* or PTH or PTB or bleed* or bled or pain* or complication*).mp | 4970897 |
| 4 | 1 and 2 and 3 | 310 |

*Note:* Search conducted on 6 August 2024.

Appendix B. IC80 ratio of COX-2 selectivity of NSAIDs included in full text review studies

| COX-1 selective | IC80 ratio | COX-2 selective | IC80 ratio | Non-selective | IC80 ratio |
| --- | --- | --- | --- | --- | --- |
| Flurbiprofen | 0.02^14^ | Mefenamic acid | >20^14^ | Ibuprofen | 0.4^14^ |
| Tenoxicam | <0.1^54^ | Etodolac | 23.3^14^ | Ketoprofen | 0.2^14^ |
| Ketorolac | <0.1^14^ | Etoricoxib | >50^55^ | Naproxen | 0.3^56^ |
| Lornoxicam | <0.1^57^ | Celecoxib | 9.1^14^ | Diclofenac | 4.3^14^ |
|  |  | Rofecoxib | >200^14^ | Aspirin | 0.3^14^ |
|  |  | Parecoxib | 110^58^ |  |  |
|  |  | Nimesulide | 5.9^14^ |  |  |

Appendix C. Critical appraisal summary

|  | Internal validity bias related to: | | | | | | | | | | | | | |
| --- | --- | --- | --- | --- | --- | --- | --- | --- | --- | --- | --- | --- | --- | --- |
|  | Domain | Selection and allocation | | | Administration of intervention/exposure | | | Assessment, detection and measurement of the outcome | | | Participant retention | Statistical conclusion validity | | |
|  | Question number | 1 | 2 | 3 | 4 | 5 | 6 | 7 | 8 | 9 | 10 | 11 | 12 | 13 |
| Study | Outcome |  |  |  |  |  |  |  |  |  |  |  |  |  |
| Ng, 2017 | Readmissions for analgesia | Y | Y | Y | Y | Y | Y | Y | Y | Y | Y | Y | Y | Y |
|  | Average number of doses of PRN analgesia |  |  |  |  |  |  | Y | Y | N | Y | Y | Y |  |
|  | PONV |  |  |  |  |  |  | Y | Y | Y | Y | Y | Y |  |
| Xie, 2012 | Primary bleeding | Y | Y | Y | Y | Y | Y | Y | Y | Y | Y | Y | Y | Y |
|  | Number of patients needing PRN analgesia |  |  |  |  |  |  | Y | Y | Y | Y | Y | Y |  |
|  | PONV |  |  |  |  |  |  | Y | Y | Y | Y | Y | Y |  |
| Joshi, 2003 | Primary bleeding | U | U | U | U | U | U | U | U | Y | U | Y | Y | U |
|  | Average number of doses of PRN analgesia |  |  |  |  |  |  | U | U | Y | Y | Y | N |  |
|  | PONV |  |  |  |  |  |  | U | U | U | U | Y | Y |  |
| Van Daele, 2016 | Primary bleeding | Y | Y | Y | Y | Y | Y | Y | Y | N | Y | Y | Y | Y |
|  | Secondary bleeding |  |  |  |  |  |  | Y | Y | N | Y | Y | Y |  |
|  | Overall bleeding |  |  |  |  |  |  | Y | Y | N | Y | Y | Y |  |
|  | Readmissions for analgesia |  |  |  |  |  |  | Y | Y | N | Y | Y | Y |  |
|  | Number of patients needing PRN analgesia |  |  |  |  |  |  | Y | Y | N | Y | Y | Y |  |
|  | PONV |  |  |  |  |  |  | Y | Y | N | Y | Y | Y |  |
| Murto, 2015 | Overall bleeding | Y | Y | Y | Y | Y | Y | Y | Y | N | Y | Y | Y | Y |
|  | Average number of doses of PRN analgesia |  |  |  |  |  |  | Y | Y | N | Y | Y | Y |  |
|  | PONV |  |  |  |  |  |  | Y | Y | N | Y | Y | Y |  |
|  | Abdominal pain |  |  |  |  |  |  | Y | Y | N | Y | Y | Y |  |
| Giordano, 2023 | Primary bleeding | Y | Y | Y | Y | Y | Y | Y | Y | N | Y | Y | Y | Y |
|  | Secondary bleeding |  |  |  |  |  |  | Y | Y | N | Y | Y | Y |  |
|  | Overall bleeding |  |  |  |  |  |  | Y | Y | N | Y | Y | Y |  |
|  | Average number of doses of PRN analgesia |  |  |  |  |  |  | Y | Y | N | Y | Y | Y |  |
|  | PONV |  |  |  |  |  |  | Y | Y | N | Y | Y | Y |  |
|  | Abdominal pain |  |  |  |  |  |  | Y | Y | N | Y | Y | Y |  |
| Nikanne, 2005 | Primary bleeding | Y | Y | Y | Y | Y | Y | U | Y | U | Y | N | Y | N |
|  | Transfusion |  |  |  |  |  |  | U | Y | U | Y | Y | Y |  |
| Sheeran, 2016 | Primary bleeding | U | U | Y | Y | U | Y | U | Y | Y | Y | Y | Y | U |
|  | PONV |  |  |  |  |  |  | U | Y | Y | Y | Y | Y |  |
| Louizos, 2006 | Primary bleeding | U | N | U | N | N | Y | N | Y | Y | Y | Y | Y | U |
|  | Number of patients needing PRN analgesia |  |  |  |  |  |  | N | Y | Y | Y | Y | Y |  |
|  | PONV |  |  |  |  |  |  | N | Y | N | Y | Y | N |  |
|  | Gastrointestinal bleeding |  |  |  |  |  |  | N | Y | N | Y | Y | Y |  |
|  | Abdominal pain |  |  |  |  |  |  | N | Y | N | Y | Y | Y |  |
| Aho, 2003 | Primary bleeding | Y | Y | Y | Y | Y | Y | Y | Y | Y | Y | Y | Y | Y |
|  | Secondary bleeding |  |  |  |  |  |  | Y | Y | Y | Y | Y | Y |  |
|  | Overall bleeding |  |  |  |  |  |  | Y | Y | Y | Y | Y | Y |  |
|  | Transfusion |  |  |  |  |  |  | Y | Y | Y | Y | Y | Y |  |
|  | Average number of doses of PRN analgesia |  |  |  |  |  |  | Y | Y | N | Y | Y | Y |  |
|  | Number of patients needing PRN analgesia |  |  |  |  |  |  | Y | Y | N | Y | Y | Y |  |
|  | Gastrointestinal bleeding |  |  |  |  |  |  | Y | Y | N | Y | Y | Y |  |
|  | Abdominal pain |  |  |  |  |  |  | Y | Y | N | Y | Y | Y |  |
| Naesh, 2005 | PONV | Y | Y | Y | Y | Y | Y | U | Y | Y | Y | Y | Y | Y |
| Li, 2016 | Primary bleeding | Y | Y | Y | Y | Y | Y | Y | Y | Y | Y | Y | Y | Y |
|  | Number of patients needing PRN analgesia |  |  |  |  |  |  | Y | Y | Y | Y | Y | Y |  |
|  | PONV |  |  |  |  |  |  | Y | Y | Y | Y | Y | Y |  |
| Lee, 2004 | Number of patients needing PRN analgesia | Y | U | U | N | N | Y | U | Y | U | U | Y | N | U |
|  | PONV |  |  |  |  |  |  | U | Y | U | U | Y | Y |  |
|  | Abdominal pain |  |  |  |  |  |  | U | Y | U | U | Y | Y |  |
| Lestiansky, 2011 | Number of patients needing PRN analgesia | U | Y | Y | Y | Y | Y | Y | Y | Y | U | Y | Y. | Y |

*Note:* Responses to critical appraisal questions are coded as: Y = Yes, U = Unclear and N = No.

Abbreviations: PONV = post-operative nausea and vomiting; PRN = as needed; Q1 = True randomisation; Q2 = Concealed allocation; Q3 = Baseline similarity; Q4 = Participant blinding; Q5 = Surgeon/Doctor blinding; Q6 = Similarity of care apart from intervention; Q7 = Assessor blinding; Q8 = Similarity of measurement; Q9 = Reliability of outcome measurement; Q10 = Complete follow-up; Q11 = Intention-to-treat analysis; Q12 = Appropriateness of statistical analysis; Q13 = RCT appropriateness

Appendix D: Risk of bias GRADE tables
